# Impact of GnRH agonist trigger on subsequent follicular phase length in ART cycles

**DOI:** 10.1007/s10815-025-03503-8

**Published:** 2025-05-09

**Authors:** Roza Berkovitz-Shperling, Yaara Libai, Shir Aviv, Ben-Yosef Dalit, Azem Foad, Feferkorn Ido

**Affiliations:** Department of Obstetrics and Gynecology, Lis Hospital for Women’s Health, Tel Aviv Sourasky Medical Center, Tel Aviv, Israel, affiliated to the Faculty of Medicine, Tel Aviv University, 6 Weizman St., 6423906 Tel Aviv, Israel

**Keywords:** GnRH agonist trigger, Follicular phase length, Ovulation trigger, PGT-M, Frozen embryo transfer, Subsequent menstrual cycle

## Abstract

**Research question:**

Does the use of GnRH agonist trigger versus hCG trigger affect the length of the subsequent follicular phase in women?

**Design:**

A retrospective cohort study analyzing 196 women undergoing controlled ovarian stimulation with freeze-all for PGT-M at a university-affiliated fertility center; 132 received GnRH agonist trigger, and 64 received hCG trigger.

**Results:**

The GnRH agonist group demonstrated a significantly longer subsequent follicular phase compared to the hCG group (18.98 ± 3.54 vs. 16.06 ± 3.13 days, *P* < .001), with extended follicular phase occurring in 90.2% versus 60.9% of cycles (*P* < .001). Both groups had comparable antral follicle counts (14.52 ± 7.71 vs. 13.00 ± 15.36, *P* = .748). Multiple regression analysis identified GnRH agonist trigger as a significant independent predictor of subsequent follicular phase length (coefficient = 4.552, 95% CI: 3.058–6.045, *P* < .001), along with BMI (coefficient = 0.188, 95% CI: 0.019–0.357, *P* = .030). The model explained 31.4% of the variance in follicular phase length (F = 7.516, *P* < .001). After adjusting for confounding variables, pregnancy rates were comparable between groups (OR = 1.763, 95% CI: 0.798–3.505, *P* = .173).

**Conclusions:**

The GnRH agonist trigger prolongs the subsequent follicular phase compared to the hCG trigger without compromising pregnancy rates. BMI showed a statistically significant but modest association with follicular phase length that requires further validation. These findings have important implications for optimizing the timing of frozen embryo transfer in subsequent cycles and may facilitate more personalized monitoring protocols.

## Highlights

GnRH agonist trigger prolongs the subsequent follicular phase by approximately five days compared to the hCG trigger without affecting pregnancy rates. This finding has important implications for FET cycle planning.

## Introduction

The physiological regulation of the menstrual cycle involves intricate feedback mechanisms between the hypothalamus, pituitary, and ovaries, with gonadotropin-releasing hormone (GnRH) playing a significant role in this complex interplay [1, 2]. This understanding has enabled the development of targeted interventions in assisted reproductive technology (ART), enabling control of the axis to prevent premature ovulation and trigger the crucial phase of final oocyte maturation [3]. Historically, human chorionic gonadotropin (hCG) was the only option available for triggering final oocyte maturation; however, GnRH agonist triggers have emerged as a significant advancement in this field, offering an effective alternative to hCG [4, 5].

The primary clinical application of GnRH agonist triggers is preventing ovarian hyperstimulation syndrome (OHSS), an iatrogenic complication of controlled ovarian stimulation characterized by increased capillary permeability and potential fluid accumulation [6, 7]. By inducing an endogenous LH surge through activation of the GnRH receptor, these agents effectively promote oocyte maturation while minimizing OHSS risk in susceptible patients [8, 9]. Their impact extends beyond the immediate trigger phase, affecting various aspects of the reproductive cycle [10, 11].

A distinctive feature of GnRH agonist triggers is their effect on luteal phase dynamics. In contrast to hCG's sustained luteotropic support, GnRH agonist triggers initiate luteolysis through dual mechanisms: direct apoptotic effects on granulosa-luteal cells and a shortened LH surge [12, 13]. This process exhibits significant individual variation in timing and intensity [14], with consequent effects on implantation and pregnancy rates in fresh embryo transfer cycles [15]. Although modified luteal support protocols have been developed to address these effects [16], the broader implications for cycle dynamics remain under investigation [17].

Of particular interest is the post-trigger hypogonadotropic state induced by GnRH agonist administration [18]. This temporary perturbation of the hypothalamic-pituitary-ovarian axis may influence subsequent follicular phase characteristics [19]. Despite extensive research on immediate post-trigger effects, limited data exist regarding their impact on subsequent cycle parameters, particularly follicular phase length [20, 21]. Understanding these temporal relationships is clinically relevant for optimizing frozen embryo transfer timing and overall treatment outcomes [22, 23].

The present study investigates the influence of GnRH agonist triggers on subsequent follicular phase length, addressing a knowledge gap in reproductive endocrinology. This research aims to enhance our understanding of post-trigger cycle dynamics and inform evidence-based protocol optimization for frozen embryo transfer cycles.

## Materials and methods

### Study design and setting

This retrospective cohort study was conducted at a single academic fertility center between January 2020 and March 2023. The institutional review board approved the study (approval number TLV-0149–20) and waived the requirement for informed consent due to the study's retrospective nature.

### Study population

Women who underwent controlled ovarian stimulation (COS) between January 2020 and March 2023 were screened for eligibility. To ensure group homogeneity and minimize potential confounding factors, the analysis was limited to women undergoing COS for preimplantation genetic testing for monogenic diseases (PGT-M). Women were included if they received either a GnRH agonist or hCG trigger for final oocyte maturation. They had a modified natural frozen embryo transfer cycle in the immediate subsequent cycle. Exclusion criteria included irregular cycles, use of oral contraceptives, or inability to provide documented evidence of at least three regular menstrual cycles before treatment initiation.

### Ovarian stimulation protocol

All patients underwent an antagonist protocol using either recombinant FSH (150–300 IU/day; Gonal-F®, Merck Healthcare, Darmstadt, Germany), recombinant FSH with LH (150–300 IU/day; Pergoveris®, Merck Healthcare), or highly purified hMG (150–300 IU/day; Menopur®, Ferring Pharmaceuticals). GnRH antagonist (0.25 mg/day; Cetrotide® or Orgalutran®) was initiated when the lead follicle reached 13 mm. Final oocyte maturation was achieved using either GnRH agonist (0.2 mg triptorelin; Decapeptyl®, Ferring) or recombinant hCG (250 μg; Ovitrelle®, Merck). The choice of trigger was based on ovarian response and OHSS risk assessment, with GnRH agonist preferred for patients with high ovarian response (> 15 follicles ≥ 12 mm). Oocyte retrieval was performed 36 h post-trigger, followed by embryo biopsy and cryopreservation.

### Modified natural cycle monitoring

Monitoring of the subsequent frozen embryo transfer cycle began with transvaginal ultrasound on cycle days 7–10, these were unmedicated, modified natural cycles. Serial ultrasounds and serum hormone (estradiol, progesterone) measurements were performed every 2–3 days. Final oocyte maturation was triggered with hCG (250 μg Ovitrelle®) when the lead follicle reached 18–20 mm and progesterone levels remained < 1.5 ng/mL. Trigger timing was optimized based on follicular development and clinical and embryologic lab scheduling considerations.

### Outcome measures

The primary outcome was the change in follicular phase length, defined as the difference in the number of days from the start of the cycle (the first day of menstrual bleeding) to ovulation in the cycles following the stimulation cycle compared to the patient's baseline follicular phase length. Pre-treatment unmedicated cycles were utilized to establish the baseline length of the follicular phase. Secondary outcomes included characteristics of the cycle and endometrial thickness. An extended follicular phase in the subsequent cycle was defined as any length that exceeded the baseline, while significant prolongation was defined as exceeding the baseline by more than two days. The total cycle duration was calculated from the first day of menstrual bleeding in the stimulation cycle to documented ovulation in the susbequent modified natural cycle. Only embryos that tested negative for the specific genetic disease being evaluated in each couple were transferred. Pregnancy was confirmed by detecting appropriately rising serum β-hCG levels in at least two consecutive measurements.

### Power calculation

Power analysis was performed using G*Power software (version 3.1.9.7, Heinrich Heine University). Based on preliminary data showing follicular phase differences between natural cycles (14.00 ± 1.54 days) and post-GnRH agonist cycles (16.00 ± 3.29 days, effect size = 0.78), we determined that 26 patients per group would achieve 80% power with α = 0.05 (two-tailed).

### Statistical analysis

Statistical analyses were performed using SPSS version 29 (IBM Corp.). The Shapiro–Wilk test was used to assess the normality of continuous variables. Normally distributed variables were presented as mean ± standard deviation and compared using Student's t-test, while non-normally distributed variables were presented as median [interquartile range] and analyzed using the Mann–Whitney U test. Categorical variables were expressed as frequencies and percentages and compared using chi-square or Fisher's exact test as appropriate. Within-group comparisons of follicular phase lengths (natural, stimulation, and subsequent cycles) were performed using paired sample t-tests. Multiple linear regression using the enter method identified independent predictors of subsequent follicular phase length, with results presented as beta coefficients with 95% confidence intervals. Model fit was assessed using adjusted R^2^ values. All statistical tests were two-sided, with *P* < 0.05 considered significant.

## Results

Of 196 eligible patients, 132 received the GnRH agonist trigger and 64 received the hCG trigger. The baseline characteristics were comparable between groups, including mean age (34.75 ± 4.72 vs. 35.10 ± 4.70 years), BMI (22.70 ± 4.24 vs. 22.59 ± 3.85 kg/m^2^), and natural follicular phase length (13.65 ± 1.58 vs. 14.09 ± 1.58 days). However, the GnRH agonist group had significantly lower baseline FSH levels (6.98 ± 2.38 vs. 8.24 ± 3.47 mIU/mL, *P* = 0.008) but comparable Antral follicle count (AFC) (14.52 ± 7.71 vs. 13.00 ± 15.36, *P* = 0.748). The GnRH agonist group received lower gonadotropin dosages (2509 ± 886 vs. 2915 ± 1162 IU, *P* = 0.015), and yielded significantly more oocytes (median [IQR]: 15.55 [13.27] vs. 4.58 [7.15], *P* < 0.001) (Table 1).

**Table 1:** Baseline characteristics of patients according to ovulation trigger type

	GNRH-a trigger at OPU (*n* = 132)	HCG-trigger at OPU (*n* = 64)	*P*-value
Demographic characteristics			
Age (y)	34.75 ± 4.72	35.10 ± 4.70	0.629
BMI (kg/m^2^) (mean ± SD)	22.70 ± 4.24	22.59 ± 3.85	0.873
Hormonal profile			
FSH (mIU/mL)	6.98 ± 2.38	8.24 ± 3.47	0.008
LH (mIU/mL)	5.69 ± 4.34	5.21 ± 2.48	0.421
Prolactin (mIU/mL)	286.08 ± 302.45	264.98 ± 167.99	0.609
AFC	14.52 ± 7.71	13.00 ± 15.36	0.748
Follicular phase length (d)			
Natural cycle	13.65 ± 1.58	14.09 ± 1.58	0.068
COS cycle	15.32 ± 2.03	15.69 ± 2.64	0.286
Protocol characteristics			
Gonadotrophins dosage (IU)	2509 ± 886	2915 ± 1162	0.015
GnRH antagonist	132 (100%)	64 (100%)	NS
Total oocytes retrieved†	15.55 [13.27]	4.58 [7.15]	< 0.001

Values are mean ± SD or *n* (%) unless otherwise noted. *Calculated using Student's t-test for continuous variables and chi-square test for categorical variables †Values are median [interquartile range]. Abbreviations: *BMI* Body mass index; *AFC* Antral follicle count; *COS* Controlled ovarian stimulation; *GnRH* Gonadotropin-releasing hormone; *hCG* human chorionic gonadotropin; *NS* Not significant; *SD* Standard deviation; *y* years; *d* days

In subsequent cycles, the GnRH agonist group exhibited a significantly longer follicular phase compared to the hCG group (18.98 ± 3.54 vs. 16.06 ± 3.13 days, *P* < 0.001). The incidence of extended follicular phases (any prolongation from baseline) was greater in the GnRH agonist group (90.2% vs. 60.9%, *P* < 0.001), as was the occurrence of phase length differences exceeding two days from baseline (75.8% vs. 37.5%, *P* < 0.001). Endometrial thickness remained comparable between groups (8.38 ± 1.66 vs. 8.46 ± 1.56 mm, *P* = 0.759). Total cycle duration was similar (47.82 ± 13.09 vs. 45.55 ± 5.08 days, *P* = 0.174), although there was greater variability in the GnRH agonist group (Table 2).

**Table 2:** Outcomes in subsequent cycles according to previous trigger type

Parameter	GNRH agonist (*n* = 132)	HCG (*n* = 64)	*P*-value
Last Endometrial thickness (mm)‡	8.38 ± 1.66	8.46 ± 1.56	0.759
Cycle characteristics			
Subsequent cycle trigger type, hCG	132 (100)	64 (100)	
Follicular phase length (d)	18.98 ± 3.54	16.06 ± 3.13	< 0.001
Extended follicular phase§	119 (90.2)	39 (60.9)	< 0.001
Phase length difference #	100 (75.8)	24 (37.5)	< 0.001
Total cycle duration (d)‖	47.82 ± 13.09	45.55 ± 5.08	0.174
Treatment outcomes			
Blastocyst transfer *n* (%)	98 (74.8)	34 (69.4)	0.468
Pregnancy¶	73/131 (55.7)	19/49 (38.8)	0.042

Values are mean ± SD or *n* (%) unless otherwise noted. *Student's t-test for continuous variables, chi-square test for categorical variables; ‡Measured obtained within 24–48 h before trigger §Defined as subsequent cycle longer than the natural cycle; # Defiened as > 2 days difference between subsequent and natural cycle length; ‖Duration from first day of OPU cycle to ovulation in subsequent cycle ¶Denominator represents number of embryo transfers performed. Abbreviations: *d* days; *GnRH* Gonadotropin-releasing hormone; *hCG* human chorionic gonadotropin; *mm* millimeters; *NS* Not significant; *OPU* Oocyte pick-up; *pg/mL* picograms per milliliter; *SD* Standard deviation

Paired sample analysis demonstrated a significant lengthening of the follicular phase in the GnRH agonist group compared to both the patient's natural cycles (−5.33 ± 3.83 days, *P* < 0.001) and stimulation cycles (−3.66 ± 3.79 days, *P* < 0.001). The hCG group showed minimal variation between stimulation and subsequent cycles (−0.37 ± 4.06 days, *P* = 0.464) (Table 3).

**Table 3:** Comparison of follicular phase lengths between different treatment cycles*

Treatment group	Phase comparison	Follicular phase length (days)	Mean difference (SD)	95% CI	*P*-value
GNRH-a trigger at OPU (*n* = 132)	Natural vs. COS	13.65 ± 1.58 vs. 15.32 ± 2.03	−1.67 ± 2.63	−2.13 to −1.22	< 0.001
	Natural vs. Subsequent	13.65 ± 1.58 vs. 18.98 ± 3.54	−5.33 ± 3.83	−5.94 to −4.67	< 0.001
	COS vs. Subsequent	15.32 ± 2.03 vs. 18.98 ± 3.54	−3.66 ± 3.79	−4.32 to −3.09	< 0.001
HCG-trigger at OPU (*n* = 64)	Natural vs. COS	14.09 ± 1.58 vs. 15.56 ± 2.64	−1.59 ± 3.14	−2.38 to −0.81	< 0.001
	Natural vs. Subsequent	14.09 ± 1.58 vs. 16.06 ± 3.13	−1.96 ± 3.46	−2.83 to −1.10	< 0.001
	COS vs. Subsequent	15.56 ± 2.64 vs. 16.06 ± 3.13	−0.37 ± 4.06	−1.39 to 0.64	0.464

^*^Values are presented as mean ± standard deviation †Negative values indicate an increase in phase length

Abbreviations: *CI* Confidence interval; *COS* Controlled ovarian stimulation; *GnRH* Gonadotropin-releasing hormone; *hCG* human chorionic gonadotropin; *SD* Standard deviation; *vs.* versus

Multiple linear regression analysis identified the GnRH agonist trigger as a significant independent predictor of subsequent follicular phase length (coefficient = 4.552, 95% CI: 3.058–6.045, *P* < 0.001) after controlling for confounding variables. BMI was also recognized as a significant predictor (coefficient = 0.188, 95% CI: 0.019–0.357, *P* = 0.030). The model accounted for 31.4% of the variance (F = 7.516, *P* < 0.001) (Table 4).

**Table 4:** Multiple linear regression analysis for subsequent follicular phase length*

	β coefficient (SE)	Confidence interval 95%	*P*-value
Constant	7.982 (5.299)	−2.524–18.487	**0.135**
Primary exposure			
GnRH agonist vs. hCG trigger	4.552 (0.753)	3.058–6.045	** < 0.001**
Predictors			
Gonadotrophin dosage	0.000 (0.000)	−0.001–0.000	0.334
FSH	0.060 (0.124)	0.187–0.307	0.631
Age (years)	−0.105 (0.083)	−0.269–0.060	0.209
BMI (kg/m^2^)	0.188 (0.085)	0.019–0.357	0.030
Natural follicular phase (days)	0.260 (0.183)	−0.104–0.623	0.160
COS follicular phase (d)	0.229 (0.180)	−0.129–0.586	0.209
Total oocytes retrieved (*n*)	−0.021 (0.029)	−0.078–0.037	0.481

^*^Model fit: R^2^ = 0.362; Adjusted R^2^ = 0.314; F = 7.516, *P* < 0.001

Abbreviations: *GnRH-a* Gonadotropin-releasing hormone agonist; *OPU* Oocyte pick-up; *HCG* Human chorionic gonadotropin; *BMI* Body mass index; *COS* Controlled ovarian stimulation; *SE* Standard error; *CI* Confidence interval; *d* days

The adjusted R^2^ value was 0.314, indicating that the model explained approximately 31.,4% of the variance in the length of the subsequent follicular phase. The overall model was statistically significant (F = 7.516, *P* < 0.001)

Initial analysis revealed higher pregnancy rates in the GnRH agonist group (55.7% vs. 38.8%, *P* = 0.042) and comparable blastocyst transfer rates (74.8% vs. 69.4%, *P* = 0.468). However, multivariate logistic regression indicated that this difference was not independently associated with trigger type (OR = 1.763, 95% CI: 0.798–3.505, *P* = 0.173). Blastocyst transfer emerged as the sole significant independent predictor of pregnancy (OR = 2.093, 95% CI: 1.033–4.240, *P* = 0.040) when controlling for age (OR = 0.950, 95% CI: 0.886–1.018, *P* = 0.145) and total oocytes retrieved (OR = 1.020, 95% CI: 0.993–1.049, *P* = 0.150) (Table 5).

**Table 5:** Multivariate logistic regression analysis for pregnancy*

	β coefficient (SE)	Odd ratio (OR)	95% CI fot OR	*P*-value
GnRH agonist vs. hCG trigger	0.514	1.763	0.798–3.505	0.173
Age (years)	−0.052	0.950	0.886–1.018	0.145
Total oocytes retrieved (*n*)	0.020	1.020	0.993–1.049	0.150
Blastocyst vs. cleavage stage transfer	0.739	2.093	1.033–4.240	0.040

^*^Model statistics: Hosmer–Lemeshow goodness of fit test: χ^2^ = 8.234, *P* = 0.411; Nagelkerke R^2^ = 0.162. *CI* Confidence interval; *GnRH* Gonadotropin-releasing hormone; *hCG* human chorionic gonadotropin; *OR* odds ratio; *SE* Standard error

Secondary analysis indicated that women who achieved pregnancy were younger (33.95 ± 4.62 vs. 35.38 ± 4.70 years, *P* = 0.044) and had a higher oocyte yield (15.11 ± 13.57 vs. 10.52 ± 11.65, *P* = 0.016), with no significant differences in BMI, follicular phase length, or endometrial thickness.

## Discussion

### Principal findings

This study demonstrates that the GnRH agonist trigger is associated with a prolonged subsequent follicular phase, with an increase in length of 5.33 ± 3.83 days compared to patients'natural cycles (*P* < 0.001). This prolongation occurred in 90.2% of GnRH agonist-triggered cycles compared to 60.9% of hCG triggers (*P* < 0.001) (Table 2). Multiple regression analysis confirmed the GnRH agonist trigger as an independent predictor of this prolongation (coefficient = 4.552, 95% CI: 3.058–6.045, *P* < 0.001). The model also identified BMI as statistically significant but with a modest effect size (coefficient = 0.188, 95% CI: 0.019–0.357, *P* = 0.030) and a confidence interval approaching zero, suggesting this finding requires cautious interpretation.

### Results in context

The immediate effects of GnRH agonist triggers on luteal phase dynamics are well documented, primarily through direct apoptotic effects on granulosa-luteal cells and a shortened LH surge duration [9, 10]. Our findings extend this understanding by demonstrating that changes in cycle characteristics persist beyond the immediate luteal phase, impacting subsequent follicular development (Table 3). The total cycle duration remained relatively consistent despite variations in follicular phase length. This is explained by the longer luteal phase associated with an hCG trigger, compared to a shorter luteal phase following a GnRH agonist trigger but a longer subsequent follicular phase. This observation aligns with the current understanding of the hypothalamic pulse generator and the kisspeptin signaling system, which maintain cyclic activity independent of peripheral hormonal fluctuations [1, 2]. The preservation of this underlying rhythm, despite perturbations in gonadotropin levels, suggests that central mechanisms controlling reproductive cyclicity remain intact.

The notable differences in baseline FSH levels (6.98 ± 2.38 vs. 8.24 ± 3.47 mIU/mL, *P* = 0.008) and gonadotropin dosages (2509 ± 886 vs. 2915 ± 1162 IU, *P* = 0.015) between groups underscore potential variations in ovarian reserve, although AFC were comparable between groups (14.52 ± 7.71 vs. 13.00 ± 15.36, *P* = 0.748). These factors may influence cycle dynamics. However, multiple regression analysis indicated that the impact of the GnRH agonist trigger remained significant even after controlling for these confounding variables, reinforcing the strength of our primary finding.

Our findings reinforce recent evidence regarding the relationship between follicular phase length and reproductive outcomes. While earlier studies indicated that deviations from the physiological follicular phase duration might negatively influence success rates [12], newer evidence suggests a greater adaptability of the reproductive system [13]. Despite a significant extension of the follicular phase following the GnRH agonist trigger (mean increase of 5.33 ± 3.83 days), pregnancy rates remained comparable after adjusting for confounding variables (OR = 1.763, 95% CI: 0.798–3.505, *P* = 0.173). A similar endometrial thickness between groups (8.38 ± 1.66 vs. 8.46 ± 1.56 mm, *P* = 0.759) demonstrates adequate endometrial development preservation. These findings advocate a more flexible approach to cycle monitoring in assisted reproduction while sustaining treatment efficacy.

### Clinical and research implications

Our findings have practical implications for the planning and timing of frozen embryo transfer cycles following GnRH agonist triggers. Based on our results, clinicians should anticipate a prolonged follicular phase (mean increase of 5.33 days) in subsequent natural or modified natural cycles after a GnRH agonist trigger. This knowledge can guide monitoring schedules, suggesting that initial ultrasound monitoring could begin later in these cycles, potentially reducing unnecessary early visits. Notably, despite the difference in follicular phase length, pregnancy rates were similar between groups after controlling for confounding variables, indicating that this prolongation does not adversely affect clinical outcomes.

The identification of BMI as a statistically significant predictor of follicular phase length (coefficient = 0.188, 95% CI: 0.019–0.357, *P* = 0.030) should be interpreted with caution. Although it reached statistical significance, the small coefficient suggests a modest clinical effect—approximately a 0.2-day increase in follicular phase length per BMI unit. This association, while intriguing, requires validation in larger prospective studies before it can inform clinical practice. The finding potentially aligns with literature on metabolic influences on reproductive cycles, but the marginal confidence interval (lower bound 0.019) indicates borderline significance. Further research is needed to determine whether this represents a clinically meaningful relationship or a statistical finding with limited practical relevance.

From a research perspective, these findings enhance our limited understanding of menstrual cycle regulation. Maintaining regular cyclicity despite observed temporal shifts offers valuable insights into the underlying reproductive endocrinology. Moreover, women undergoing GnRH agonist trigger protocols represent a unique population for studying subtle changes in hypothalamic-pituitary-ovarian axis function while preserving overall reproductive competence. This cohort may serve as a significant model for future investigations into the cyclical regulation of reproductive function.

### Strengths and limitations

The study's primary strengths include its focus on a specific clinical scenario, comprehensive analysis of cycle dynamics through multiple phases, and robust statistical methodology, including multivariate analysis to control for confounding factors. The model's adjusted R^2^ value of 0.314 indicates that our measured variables can explain approximately 31.4% of the subsequent follicular phase length variance, suggesting a reasonable explanatory power for a biological system with complex multifactorial regulation.

However, several limitations must be considered. First, the study's retrospective nature and single-center design may limit its generalizability. Second, potential selection bias is indicated by significant differences in baseline characteristics between groups, particularly in FSH levels, gonadotropin dosages, and oocyte yield (15.55 vs. 4.58, *P* < 0.001). While we endeavored to control for these differences through multivariate analysis, unmeasured confounders may still affect our results. Third, the small sample size, especially in the hCG group (*n* = 64), limits statistical power for subgroup analyses and increases the risk of type II errors.

Additionally, our study focused solely on PGT-M cycles to maintain a uniform population; however, this limits applicability to other ART indications. We acknowledge that including detailed ovarian reserve markers (such as AMH and antral follicle count), comprehensive hormonal profiles during stimulation, and embryo quality parameters would enhance our analysis. The absence of these variables represents a limitation that should be addressed in future prospective studies.

### Considerations for clinical application

Several important considerations should be considered when applying this study's findings to clinical practice. The single-center, retrospective design inherently limits the generalizability of our results to other clinical settings and patient populations. Our focus on PGT-M cycles, while providing a relatively homogeneous study population, may not reflect outcomes in general IVF populations or those with different indications for treatment. The marked difference in oocyte yield between groups suggests an underlying selection bias in trigger choice, which could influence subsequent cycle characteristics.

Clinicians should note that our findings may not apply to patients with irregular cycles or those using oral contraceptives, as these populations were explicitly excluded from our analysis. Furthermore, while we demonstrated statistical significance in follicular phase prolongation, the clinical significance of a five-day difference requires further validation through prospective studies. The interpretation and application of these findings should be tailored to individual patient characteristics and clinical contexts, considering the potential impact on treatment planning and resource allocation.

### Future directions

Prospective studies with larger sample sizes and diverse patient populations are essential to validate our findings. These studies should include thorough hormonal profiling throughout the controlled ovarian stimulation and subsequent cycles to clarify the underlying endocrine mechanisms. Investigating additional variables, such as detailed ovarian reserve parameters, embryo quality, and embryo genetic testing results, would offer a complete understanding of the factors influencing cycle dynamics and reproductive outcomes.

Additionally, research examining whether modification of frozen embryo transfer protocols based on these findings could improve success rates would be valuable. Exploration of potential differences in response based on patient phenotypes (e.g., PCOS, diminished ovarian reserve) may facilitate more personalized approaches to cycle management following different trigger types.

## Conclusion

The GnRH agonist trigger significantly prolongs the subsequent follicular phase while preserving underlying reproductive rhythmicity. BMI showed a statistically significant but clinically modest association with the degree of follicular phase prolongation, representing the difference between the baseline and subsequent cycles after trigger administration, which requires further validation. Despite these temporal shifts, reproductive outcomes remain comparable between trigger types after controlling for confounding variables, though our sample size limits definitive conclusions. This knowledge contributes to our understanding of reproductive endocrinology and suggests potential implications for clinical practice, particularly regarding the timing of monitoring protocols following different trigger types. However, these findings should be interpreted cautiously and not yet broadly implemented until confirmed by prospective studies with larger, more diverse cohorts. Further research is essential to validate these preliminary observations and determine their true clinical significance and applicability across different patient populations.

## Data Availability

The datasets generated and analyzed during the current study are not publicly available due to privacy and ethical restrictions. Still, they are available from the corresponding author upon reasonable request, subject to institutional approval and data protection regulations. All authors have read and approved the final manuscript.
